# Glioma stem cells-derived exosomes promote the angiogenic ability of endothelial cells through miR-21/VEGF signal

**DOI:** 10.18632/oncotarget.16661

**Published:** 2017-03-29

**Authors:** Xu Sun, Xiaotang Ma, Jinju Wang, Yuhui Zhao, Yue Wang, Ji C. Bihl, Yanfang Chen, Chuanlu Jiang

**Affiliations:** ^1^ Department of Neurosurgery, The Second Affiliated Hospital, Harbin Medical University, Harbin 150086, China; ^2^ Guangdong Key Laboratory of Age-Related Cardiac and Cerebral Diseases, Institute of Neurology, Affiliated Hospital of Guangdong Medical University, Zhanjiang 524000, China; ^3^ Department of Pharmacology & Toxicology, Boonshoft School of Medicine, Wright State University, Dayton, OH 45435, USA; ^4^ Department of Neurology and Stroke Center, The First Affiliated Hospital, Sun Yat-Sen University, Guangzhou 510000, China

**Keywords:** GSCs, exosomes, miR-21, VEGF, angiogenesis

## Abstract

Glioma stem cells (GSCs) play an important role in glioblastoma prognosis. Exosomes (EXs) mediate cell communication by delivering microRNAs (miRs). Glioblastoma has a high level of miR-21 which could upregulate vascular endothelial growth factor (VEGF) expression. We hypothesized GSC-EXs can promote the angiogenic ability of endothelial cells (ECs) through miR-21/VEGF signal. GSCs were isolated from U-251 cells with stem cell marker CD133. GSCs transfected without or with scramble or miR-21 mimics were used to produce GSC-^EXs^con, GSC-EXs^sc^ and GSC-EXs^miR-21^. Human brain ECs were co-cultured with vehicle, GSC-EXs^con^, GSC-EXs^sc^ or GSC-EXs^miR-21^ plus VEGF siRNAs (siRNA^VEGF^). After 24 hours, the angiogenic abilities of ECs were evaluated. The levels of miR-21, VEGF and p-Flk1/VEGFR2 were determined. Results showed: 1) Over 90% of purified GSCs expressed CD133; 2) The levels of miR-21 and VEGF in GSCs and GSC-EXs were up-regulated by miR-21 mimic transfection; 3) Compared to GSC-EXs^con^ or GSC-EXs^sc^, GSC-EXs^miR-21^ were more effective in elevating the levels of miR-21 and VEGF, and the ratio of p-Flk1/VEGFR2 in ECs; 4) GSC-EXsmiR-21 were more effective in promoting the angiogenic ability of ECs than GSC-EXs^con^ or GSC-EXs^sc^, which were remarkably reduced by siRNA^VEGF^ pretreatment. In conclusion, GSC-EXs can promote the angiogenic ability of ECs by stimulating miR-21/VEGF/VEGFR2 signal pathway.

## INTRODUCTION

Glioblastoma is the most common and malignant brain tumor in adults. Current therapies for glioblastoma include surgical resection, radiotherapy and chemotherapy. However, the five-year survival rate for glioblastoma is less than 5%, and its median survival period is only 14.6 months in adults [1]. The ineffectiveness of the treatments majorly result from the tumor cellular heterogeneity, the high migratory capability of glioblastoma and chemo-resistance [2].

Cancer stem cells have been suggested to participate in tumor growth and radio- or chemo- resistance [3, 4]. Glioma stem cells (GSCs) are multipotent tumor-initiating cells which display stem cell properties [5, 6] and express CD133 marker [7, 8]. Increasing evidence suggests that the aggressiveness and unresponsiveness of glioblastoma might be related to the presence of GSCs [9, 10]. Indeed, GSCs can promote tumor angiogenesis [11] and have been shown to be resistant to cell death following growth factors withdrawal [12, 13]. Thus, understanding the mechanism of how GSCs promote metastasis of glioblastoma will be important for developing efficient therapeutic strategies.

Angiogenesis and extensive invasion are hallmark features of malignant glioblastoma. As we know, cancer cells can subvert surrounding normal cells such as endothelial cells (ECs) to promote tumor growth, angiogenesis and metastases [14, 15]. Currently, extracellular exosomes (EXs) are emerging as novel intercellular communicators. Mounting evidence has shown that EXs can convey cargoes such as proteins and microRNAs (miRs) to distant/nearby cells and modulate recipient cell function [16–18]. Exosomal miR-135b from hypoxia-resistent multiple myeloma cells could enhance endothelial tube formation under hypoxia [19]. The extracellular vesicles released from glioblastoma cells have been shown to transfer RNAs to brain microglia/macrophages [20], suggesting the potential of glioblastoma in manipulating its environment. Nevertheless, no study has investigated the potential role of GSC-derived EXs (GSC-EXs) on EC functions.

As an oncomiR, miR-21 is expressed in a wide range of cancers. It promotes cell proliferation and migration [2]. Earlier studies have identified that vascular endothelial growth factor (VEGF) plays a key role in the angiogenesis process of astrocytoma [21, 22]. Overexpressing miR-21 increased the expression of VEGF in a prostate cell line and induced tumor angiogenesis [23]. Downregulation of miR-21 in glioblastoma cells caused repression of cell growth which theoretically could enhance the chemotherapeutic effects of cancer therapy [24]. Recently, another study revealed that GSCs can produce higher levels of VEGF and contribute to tumor angiogenesis [11]. However, whether EXs-derived from GSCs (GSC-EXs) can promote the angiogenesis through their carried miRs and proteins remains unknown.

In this study, we aimed to investigate whether GSC-EXs can promote the angiogenic function of ECs through the miR-21/VEGF signal.

## RESULTS

### Purification of GSCs from U251 cells

**Figure 1:**
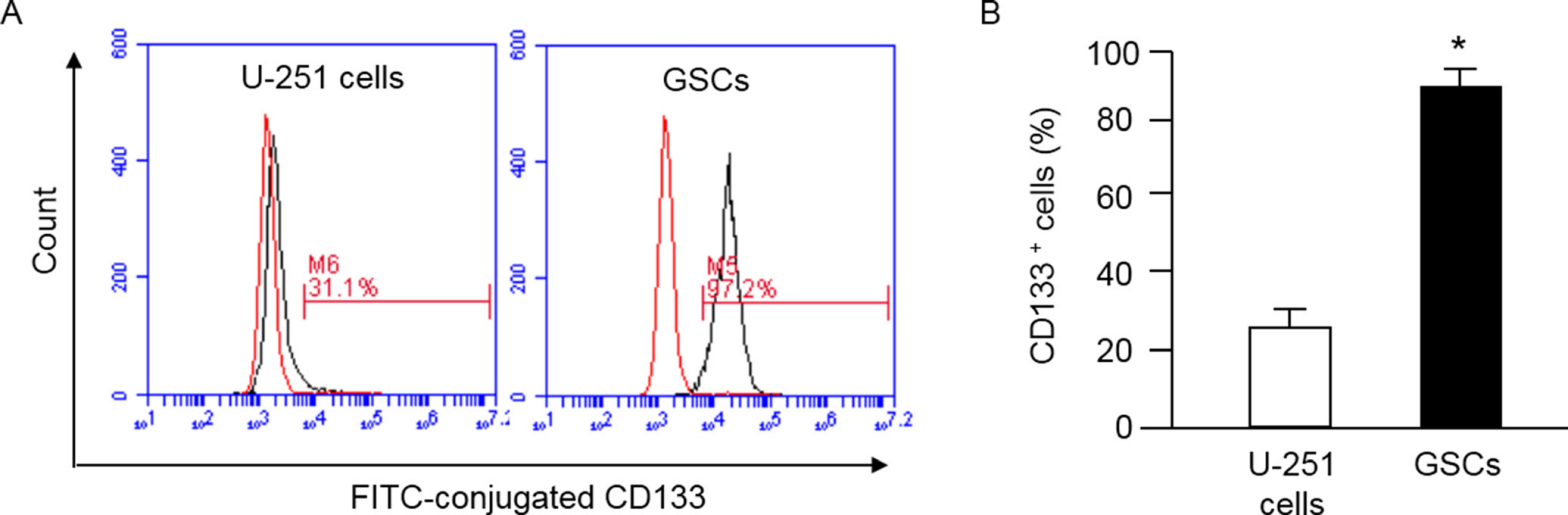
MACS purification of GSCs from U-251 cells and characterization of GSCs by flow cytometry GSCs were defined as CD133^+^ cells, which were separated by using MACS and assessed by flow cytometry for determining the efficiency of purification. (**A**) representative flow plots showing the percentage of CD133^+^ cells in U-251 cells and the purified GSCs; left curve: isotype control; right curve: antibody; (**B**) summarized data showing the percentage of CD133^+^ cells before and after MACS; *p < 0.05, vs. U-251 cells. Data are expressed as mean ± SEM; *n* = 3/group. MACS: magnetic activated cell sorting.

The cell surface antigen CD133 is considered as a marker of stem cells. Indeed, several studies have demonstrated CD133 can be used to isolate a population of cells with stem-cell properties [25, 26]. Here, we used anti-CD133 conjugated beads to isolate GSCs from U251 cells. The purification efficiency was assessed by flow cytometry. The data (Figure 1) showed that anti-CD133-conjugated beads enriched CD133^+^ cells to 90 ± 6% from U-251 cells (25 ± 5%).

### MiR-21 overexpression increased VEGF mRNA level in GSCs

**Figure 2:**
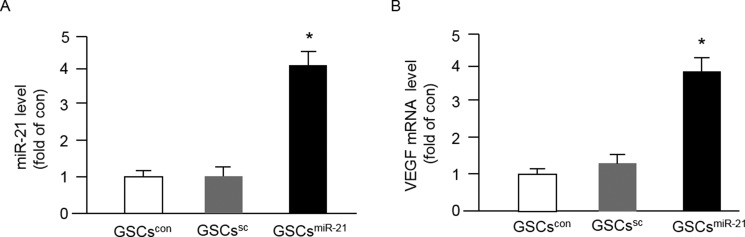
Up-regulation of miR-21 by miR-21 mimic transfection increased VEGF mRNA expression in GSCs The purified GSCs were transfected with miR-21 for 48 hrs, and the levels of miR-21 and VEGF were determined by qRT-PCR. (**A**–**B**) the levels of miR-21 and VEGF in different types of GSCs; *p < 0.05, vs. GSCs^con^ or GSCs^sc^; Data are expressed as mean ± SEM; *n* = 4/group.

The success of miR-21 mimic or inhibitor transfection was confirmed by qRT-PCR. As shown in Figure 2A, the level of miR-21 in GSCs was remarkably up-regulated by miR-21 mimics. There was no significant difference of the miR-21 level between GSCs in control (GSCs^con^) and those transfected with scramble control (GSCs^sc^). In addition, we found that miR-21 mimics elevated the mRNA level of VEGF in GSCs as compared to that in GSCs^con^ or GSCs^sc^ (Figure 2B).

### GSC-EXs^miR-21^ harbored high levels of miR-21 and VEGF

**Figure 3:**
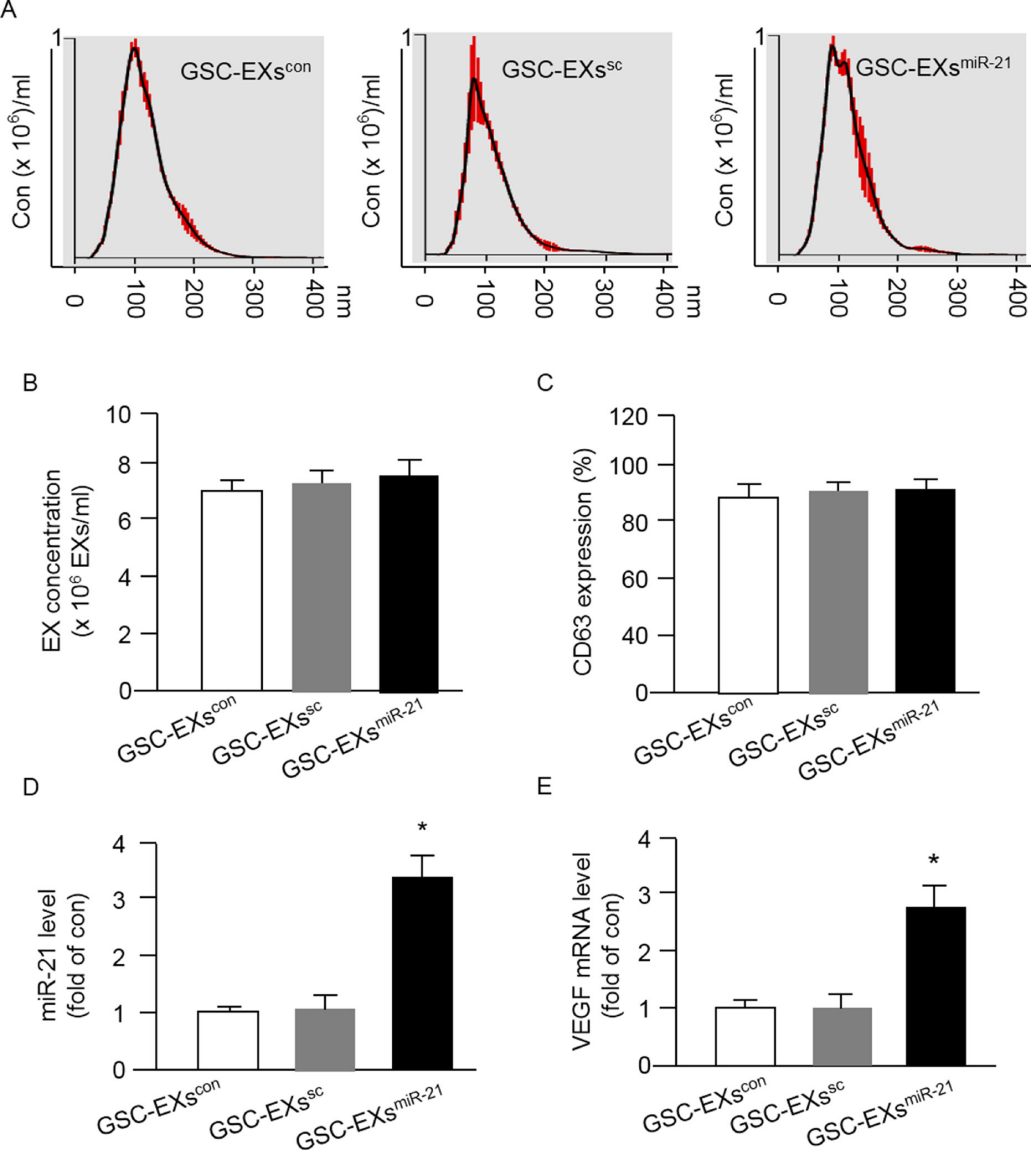
Up-regulation of miR-21 in GSCs by miR-21 mimic transfection increased the levels of miR-21 and VEGF mRNA expression in GSC-EXsmiR-21 (**A**) representative NTA plots showing the same pattern of size distribution of the three types of GSC-EXs; (**B**) summarized data showing the concentration of EXs. (**C**) CD63 expression in the collected EXs. *p < 0.05, vs. GSCs; (**D**–**E**) summarized data showing the levels of miR-21 and VEGF in various types of GSC-EXs. *p < 0.05, vs. GSC-EXs^con^ or GSC-EXs^sc^; Data are expressed as mean ± SEM; *n* = 4/group.

It is believed that EXs can carry the cargoes such as miRs and proteins from their parent cells. We focused on determining the level of miR-21 and VEGF in GSC-EXs. First of all, we analyzed the size and concentration of GSC-EXs and assessed the expression of EX specific marker CD63 by using NTA. As shown in Figure 3A–3B, the three types of GSC-EXs displayed a similar size distribution. The average size was approximately 20-120 nm. There was no difference of the concentration of EXs in the three groups. According to the fluorescence NTA results, over 90% of EXs expressed CD63 (Figure 3C).

Next, we examined the levels of miR-21 and VEGF in GSC-EXs. We found that the EXs-derived from GSCs transfected with miR-21 mimics (GSC-EXs^miR-21^) carried a higher level of miR-21 and VEGF as compared to that in the EXs-derived from control GSCs (GSC-EXs^con^) or the EXs-derived from GSCs transfected with scramble control (sc) of miR-21 (GSC-EXs^sc^) (Figure 3D–3E). Altogether, the levels of miR-21 and VEGF in GSC-EXs^miR-21^ appeared to be paralleled with their parent cells, GSCs^miR-21^.

### GSC-EXs^miR-21^ had better effects on elevating miR-21 level and VEGF secretion of ECs than GSC-EXs^con^

**Figure 4:**
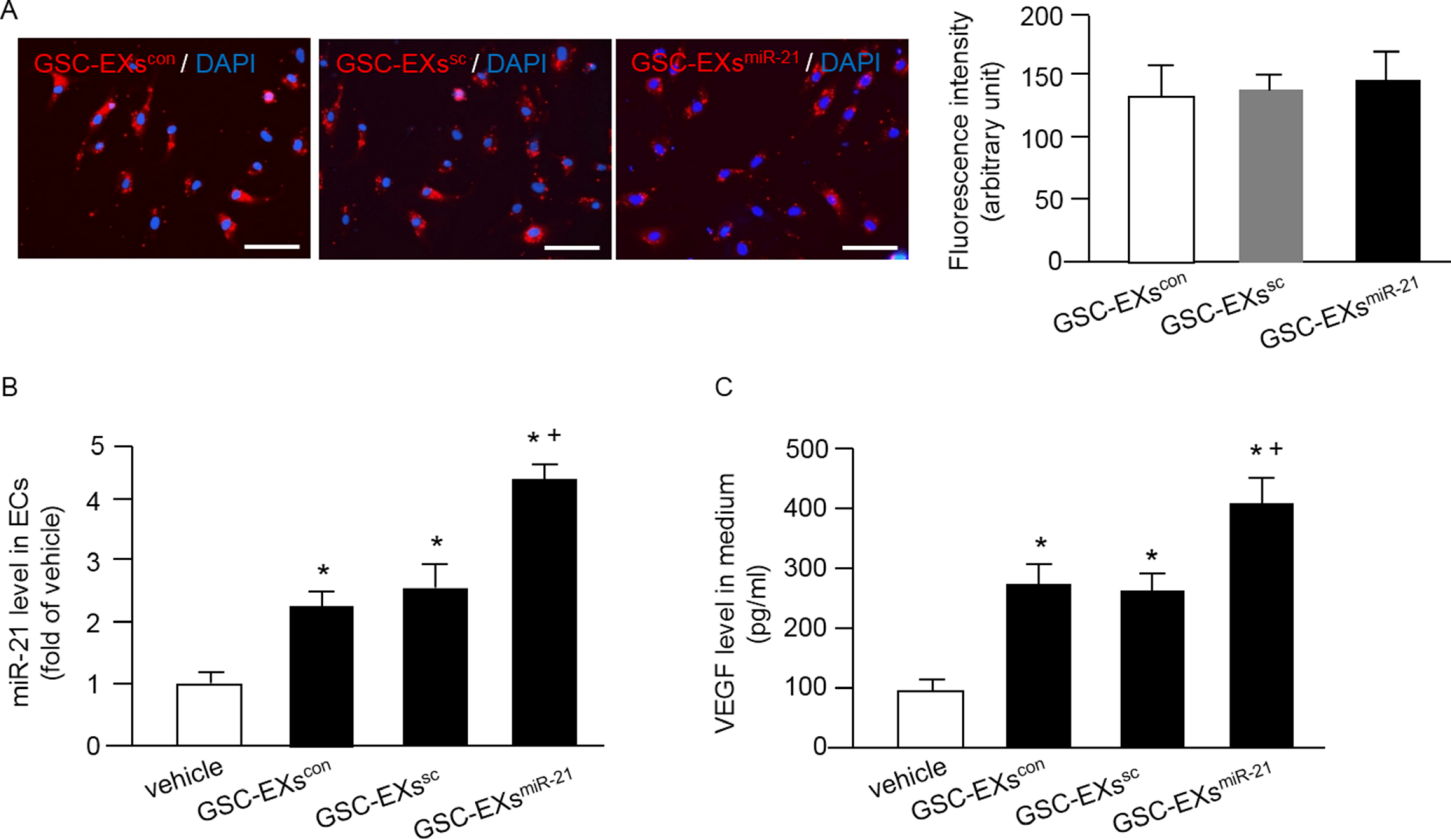
Incorporation of various GSC-EXs into ECs and GSC-EXsmiR-21 had better effects on increasing miR-21 level and VEGF secretion of ECs (**A**) left: representative images showing the incorporation of GSC-EXs into ECs; bar: 100 μm; red: PKH26 labeled GSC-EXs; blue: DAPI counterstained nucleus; right: summarized data showing the fluorescence intensity in ECs co-cultured with the three types of GSC-EXs; (**B**) summarized data showing the level of miR-21 in ECs co-cultured with the three types of GSC-EXs; (**C**) summarized data showing the concentration of VEGF in the culture medium of ECs co-cultured with GSC-EXs. *p < 0.05, vs. vehicle; ^+^*p* < 0.05, vs. GSC-EXs^con^ or GSC-EXs^sc^; Data are expressed as mean ± SEM; *n* = 4/group.

Increasing evidence shows that EXs are crucial in cell-cell communication [16, 27]. To examine whether GSC-EXs could be up-taken by ECs, the GSC-EXs were labeled with red fluorescence PKH26 and added to the culture medium of ECs. As shown in Figure 4A, the labeled GSC-EXs were observed in the cytoplasm of ECs, suggesting that the GSC-EXs can be incorporated into ECs. Meanwhile, our data showed that there was no significant difference of the fluorescence intensity in ECs after GSC-EX co-culture, indicating that similar amounts of GSC-EXs were up-taken by ECs.

Then, we assessed whether GSC-EXs can alter the levels of miR-21 and VEGF in the recipient cells, ECs. Our results (Figure 4B) showed that GSC-EXs^con^ or GSC-EXs^sc^ co-culture alone significantly increased the level of miR-21 in ECs, and this effect was further enhanced by GSC-EXs^miR-21^. These data suggest that GSC-EXs can transfer miR-21 to ECs. Similarly, a higher concentration of VEGF was detected in the culture medium of ECs co-cultured with GSC-EXs^miR-21^ than those co-cultured with vehicle (culture medium only), GSC-EXs^con^ or GSC-EXs^sc^ (Figure 4C). All of these data show that GSC-EXs^miR-21^ can up-regulate the levels of miR-21 and VEGF in ECs.

### GSC-EXs^miR-21^ had better effect than GSC-EXs^con^ on promoting the angiogenic ability of ECs

**Figure 5:**
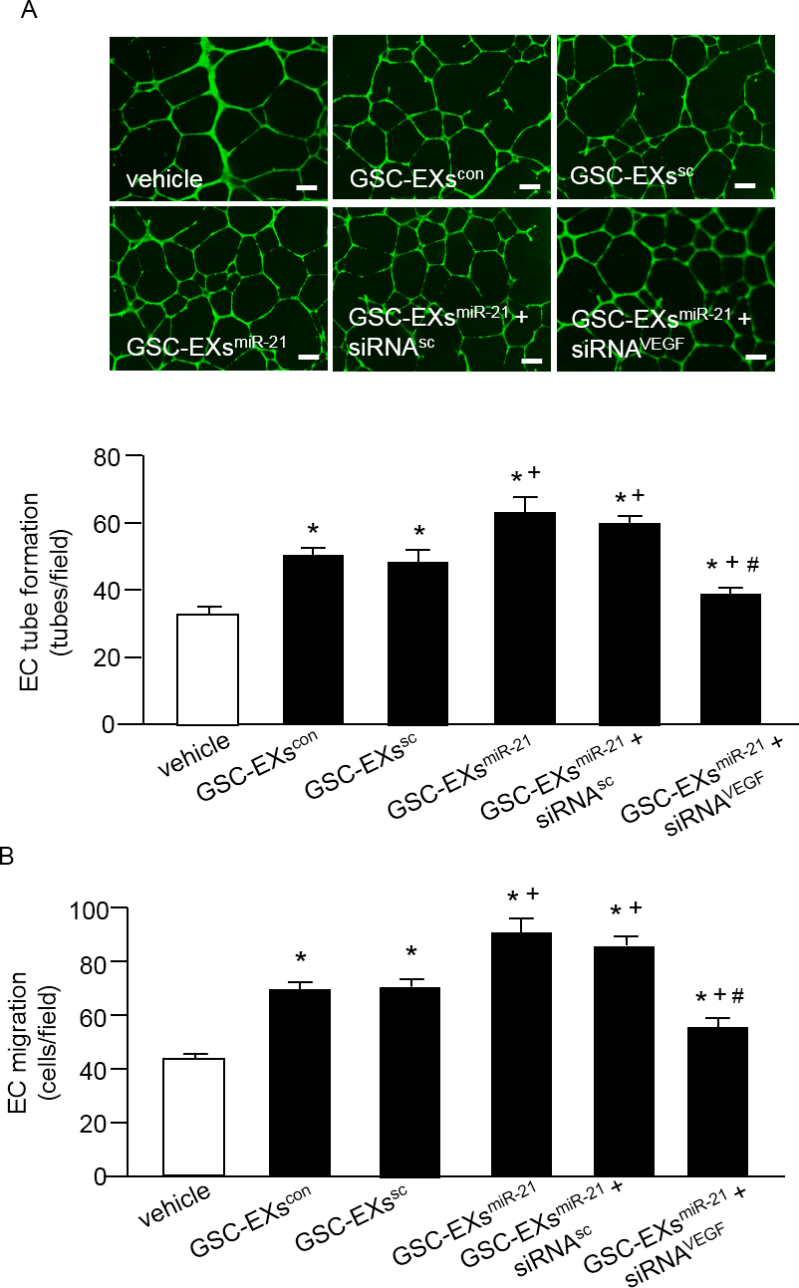
GSC-EXsmiR-21 had better effects than GSC-EXscon and GSC-EXssc on promoting the angiogenic ability of ECs (**A**) representative images and the summarized data showing the tube formation ability of ECs treated by various types of GSC-EXs; bar: 500 μm; (**B**) summarized data showing the migration ability of ECs in different treatment groups. *p < 0.05, vs. vehicle; ^+^*p* < 0.05, vs. GSC-EXs^con^ or GSC-EXs^sc^; ^#^*p* < 0.05, vs. GSC-EXs^miR-21^ or GSC-EXs^miR-21^+siRNA^sc^. Data are expressed as mean ± SEM; *n* = 4/group.

To further investigate whether miR-21 is involved in promoting the tube formation and migration abilities of ECs, we treated ECs with GSC-EXs^miR-21^ and its control, GSC-EXs^con^ or GSC-EXs^sc^ or GSC-EXs^miR-21 ko^. As shown in Figure 5, GSC-EXs^con^ or GSC-EXs^sc^ alone enhanced the tube formation and migration abilities of ECs as compared to the vehicle (culture medium only). What's more, the angiogenic ability of ECs was much greatly enhanced by GSC-EXs^miR-21^. In addition, to determine whether VEGF is involved in the effect of GSC-EXs, we blocked VEGF with VEGF siRNA (siRNA^VEGF^) in ECs. Our data showed that siRNA^VEGF^ remarkably reduced the angiogenic ability of ECs elicited by GSC-EXs^miR-21^. Altogether, these data indicate that GSC-EXs increase the angiogenic ability of ECs through miR-21/VEGF.

### GSC-EXs activated the VEGF/VEGFR2 pathway in ECs

**Figure 6:**
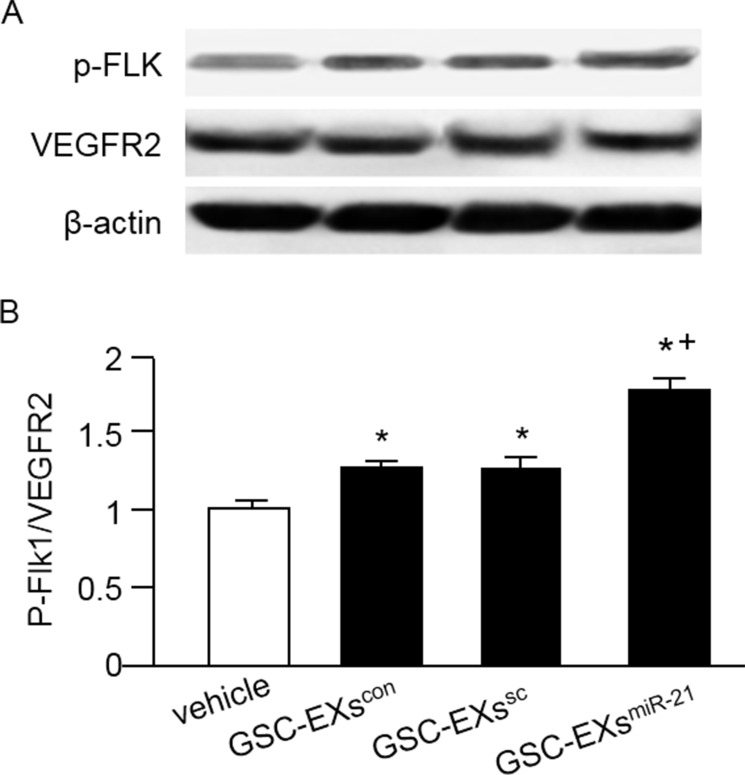
GSC-EXsmiR-21 had better effect than GSC-EXscon and GSC-EXssc on activating the VEGF/VEGFR2 signal in ECs (**A**) representative bands showing the expressions of VEGFR2 and its phosphorylation in ECs; (**B**) summarized data showing the ratio of p-Flk1/VEGFR2. * *p* < 0.05, vs. vehicle; ^+^*p* < 0.05, vs. GSC-EXs^con^ or GSC-Exs^sc^. Data are expressed as mean ± SEM; *n* = 4/group.

It is known that VEGFR2 is responsible for most downstream angiogenic effects of VEGF in tumors including migration, invasion, and endothelial proliferation. For assessing whether the GSC-EX co-culture can activate the VEGF/VEGR2 signal, we measured the phosphorylation of VEGFR2 in ECs. Our data (Figure 6) showed that both GSC-EXs^con^ and GSC-EXs^sc^ significantly raised the ratio of p-Flk/VEGFR2 in ECs, which was further enhanced by GSC-EXs^miR-21^.

## DISCUSSION

The main findings of this study include that 1) GSC-EXs can promote the angiogenic ability of ECs; 2) miR-21/VEGF/VEGFR2 signaling is the underlying mechanism of the effects elicited by GSC-EXs.

Cancer stem cells participate in tumor growth and chemotherapy resistance [3, 4]. Increasing evidence suggests that the presence of GSCs could result in the aggressiveness of glioblastoma [9, 10], although the exact mechanism of how GSCs promote metastasis of glioblastoma is unclear. CD133 has been well recognized as a marker for GSCs [25, 26]. We successfully purified GSCs from U-251 cells by using CD133-conjugated beads, with the purity > 90% as confirmed by flow cytometry (Figure 1).

In recent years, increasing evidence indicates that EXs function as intercellular messengers to facilitate cancer progression and metastasis [27, 28]. Indeed, tumor-derived EXs have been shown to modify healthy cells by altering their translational profile to promote tumor progression [29]. Extracellular vesicles derived from glioblastoma cells could modify recipient cells (endothelial or monocytes) through transferring proteins and messenger RNAs and non-coding RNAs [16–18]. In the present study, we aimed to investigate whether GSC-EXs plays a role in promoting the angiogenic ability of ECs. Our data (Figure 5) revealed that GSC-EXs^con^ offered a promotion effect on tube formation and migration ability of ECs, supporting the notion that EXs released from malignant cells can affect the function of surrounding cells.

It is known that extensive angiogenesis and invasion are two typical features of glioblastoma. The pro-angiogenic role of miR-21 has been indicated in a previous study showing that knock-down of miR-21 decreased neointimal hyperplasia following angioplasty [30]. Increasing evidence shows that the level of miR-21 is significantly elevated in human cancers include glioblastoma and melanoma [31, 32]. It is known that miRs transferred via EXs could mediate cell-cell communication, however, whether GSC-EXs can transfer miR-21 to interfere the angiogenic ability of ECs remains unknown. In order to explore the role of miR-21 in angiogenic ability of ECs promoted by GSCs-EXs, we overexpressed miR-21 in GSCs to obtain miR-21 enriched EXs (GSC-EXs^miR-21^). The miR-21 level in GSCs and the correspond EXs was verified by qRT-PCR. Our data showed that miR-21 level was elevated in GSC-EXs^miR-21^, which paralleled with their parent cells, GSCs^miR-21^ (Figures 2 and 3), suggesting the success of transfection. Previous studies have revealed that VEGF is involved in EC recruitment, proliferation and vasculature formation to support the expansion of malignant tumors [33, 34]. In this study, we found that the mRNA level of VEGF was increased in GSC-EXs^miR-21^ (Figure 2). This is in consistent with previous studies showing that miR-21 can enhance VEGF expression [23, 28].

In order to elucidate whether GSC-EXs co-culture can affect the miR-21 level and VEGF secretion of ECs, we directly cultured ECs in the presence of different types of GSC-EXs. Our results demonstrated that the PKH26 labeled GSC-EXs were up-taken by ECs after co-culture (Figure 4). More importantly, we found that the GSC-EXs^miR-21^ can remarkably increase miR-21 level in ECs, suggesting that the GSC-EXs^miR-21^ can delivery miR-21 to ECs. Meanwhile, we found the level of VEGF in the culture medium of ECs was significantly elevated after the incubation with GSC-EXs^miR-21^(Figure 4). The elevated level of miR-21 could be attributed to the directly delivery of VEGF via GSC-EXs^miR-21^ and/or through the secretion of VEGF of ECs enhanced by GSC-EXs^miR-21^. This data was supported by a previous report showing that exosomal miR-21 can activate STAT3 to increase VEGF secretion of human umbilical vein ECs [28].

For further assess whether GSC-EXs ^miR-21^ harboring high levels of miR-21 and VEGF can promote the angiogenesis response, ECs were evaluated by using tube formation assay. The results revealed that GSC-EXs^con^ and GSC-EXs^sc^ alone can promote tube formation on the matrigel, and GSC-EXs^miR-21^ exhibited the best effect (Figure 5). These data were supported by a previous report showing that the extracellular vesicles derived from U251 cells had the ability to stimulate EC activities [35]. The proved pro-angiogenic ability of GSC-EXs could be explained, at least partially, was attributed to their miR-21 and VEGF contents. VEGF is well known as a pro-angiogenic factor [36]. Its release by tumor cells as a component of membrane vesicles has been highlighted [37]. In order to verify the role of VEGF in promoting EC angiogenesis, we knocked down VEGF by using siRNA^VEGF^ and observed that a significantly less amount of tubes were formed by ECs, reflecting that VEGF is involved in GSC-EXs mediated endothelial angiogenesis.

It is believed that VEGFR2 is responsible for most downstream angiogenic effects of VEGF including changes in vascular permeability, endothelial proliferation, invasion, migration, and survival [38]. Binding of VEGF to VEGFR2 can activate downstream survival and migration pathways involving PI3-kinase/Akt and focal adhesion kinase [39]. In the present study, our results indicate that the ratio of p-Flk1/VEGFR2 was significantly elevated in ECs co-cultured with the GSC-EXs^miR-21^ (Figure 6). These data reflect that the VEGF/VEGFR2 signal was activated in ECs after the incubation with GSC-EXs.

Altogether, our results indicate that GSC-EXs fully equipped for angiogenesis stimulation through their carried miR-21 and pro-angiogenic growth factor VEGF to activate the angiogenic ability of ECs. These data suggest an important role for GSCs in tumor angiogenesis and in elucidation of the mechanistic basis which will benefit the development of novel therapeutic strategies. However, *in vivo* research should be done for further studying the effects of GSC-EXs in tumor metastasis.

## MATERIALS AND METHODS

### Cell culture

Human GBM cell line U-251 cells were purchased from ATCC (Manassas, VA, USA). The cells were cultured with DMEM medium supplemented with 4.5g/L glucose, 2 mM L-glutamine and 10% fetal bovine serum (FBS) according to the manufacture's instruction. Medium was replaced twice a week.

Human brain ECs were purchased from Cell systems (Kirkland, WA, USA) and cultured according to the manufacturer's protocol. In brief, ECs were cultured in CSC complete medium containing 10% serum, 2% human recombinant growth factors, and 0.2% antibiotic solution under standard cell culture conditions (5% CO2, 37°C). All medium and supplement reagents were purchased from Cell Systems. Medium was changed twice a week.

### Purification of GSCs with CD133-conjugated microbeads from glioblastoma cells by using magnetic activated cell sorting

CD133 has been used to enrich the putative cancer stem cells [25, 26]. In this study, the anti-CD133-conjugated microbeads were applied to isolate GSCs from U-251 cells by using magnetic activated cell sorting (MACS) as previously reported with slight modification [40]. In brief, U-251 MG cells were incubated with anti-CD133-conjugated microbeads antibody (10 μl anti-CD133 microbeads per 10^7^ U251 cells) in 100 μl reaction volume for 20 mins in the refrigerator. Then, the CD133^+^ cells were collected by using a magnet separator (DynaMag-2 magnet; Thermo scientific). The purity of GSCs was confirmed by flow cytometry analysis. The purified GSCs were expanded in DMEM/F12 medium containing 2% B27 (without retinoic acid), EGF (20 ng/ml), FGF-2 (20 ng/ml), heparin (5 μg/ml), glutamine (2 mM) and 1% antibiotics.

For flow cytometry analysis, the purified GSCs and U-251 MG cells were washed with PBS twice, and then incubated with FITC-conjugated CD133 (5 μl/1×10^6^ cells, Miltenyi Biotec), or isotype control antibody (FITC-conjugated IgG, 20 μl/1×10^6^ cells, BD biosciences) for 30 mins in the dark. After incubation, the samples were analyzed under flow cytometry (BD C6 flow cytometer). 10,000 events were collected for analysis. The experiment was repeated three times.

### Cell transfection

The purified GSCs were expanded and used for miR-21 mimics transfection to overexpress miR-21 [41]. Briefly, the GSCs were cultured to 60–70% confluence, and transfected with miR-21 mimics or the SC of miR-21 (40 nM, Thermo Fisher Scientific, Waltham, MA) by using lipofectamine 2000 (Invitrogen, Carlsbad, CA) for 48 hrs according to the manufacturer's instruction. The sequences of miR-21 mimics were: sense 5′-UAGCUUAUCAGACUGAUGUUGA-3′; antisense 5′-AACAUCAGUCUGAUAAGCUAUU-3′. GSCs transfected with miR21 SC or mimics or inhibitors were denoted as GSCs^sc^ or GSCs^miR-21^, respectively. GSCs cultured in complete culture medium served as control (GSCs^con^). The levels of miR-21 and protein in GSCs were extracted after transfection, respectively. The experiment was repeated four times. The three types of GSCs were used for producing corresponding EXs.

### Preparation and collection of EXs released from GSCs

The protocol for collecting EXs from serum-free conditionl medium (CM) has been reported in our previous study [42]. Briefly, GSCs^con^, GSCs^sc^, GSCs^miR-21 ko^ or GSCs^miR-21^ were cultured in CM composed of DMEM medium supplemented with 4.5g/L glucose, 2 mM L-glutamine to release EXs which were denoted as GSC-EXs^con^, GSC-EXs^sc^ or GSC-EXs^miR-21^. After 24 hrs, the respective CM was collected and centrifuged at 300g, 15 mins to remove dead cells. The supernatants were centrifuged at 2000 g, 30 mins to remove cell debris, followed by centrifugation at 20,000 g, 70 mins, and ultracentrifugation at 170,000 g, 90 mins to pellet EXs. The pelleted EXs were resuspended with phosphate-buffered saline (PBS) and aliquoted for nanoparticle tracking analysis (NTA) and co-culture experiments. PBS was filtered through 20 nm-filter (Whatman, Pittsburgh, PA).

### Nanoparticle tracking analysis of GSC-EXs

The NanoSight NS300 (Malvern Instruments, Malvern, UK) was used to analyze the size, concentration and CD63 expreesion of EXs at light-scatter or fluorescence-scatter mode as we previously reported [42]. Briefly, for size and concentration detection, the collected EXs were resuspended with 700 ul filtered PBS and analyzed under light-scatter mode of NTA. For detection of CD63 expression in EXs, the collected EXs were incubated with CD63-conjugated microbeads (10 ul; Miltenyi Biotec) in a 100 μl reaction volume for 2 hrs. Then, a magnet module (DynaMag-2 magnet; Life technology) was applied to separate CD63^+^ EXs from the total EX suspension. After an overnight magnet separation, the CD63^+^ EXs were resuspended with 100 μl filtered PBS and incubated with rabbit anti-goat IgG conjugated with Q-dot 655 (1:350; Life Technologies) for 90 mins at RT. All samples were analyzed under fluorescence-scatter mode of NTA. Three videos of typically 30 seconds duration were taken, with a frame rate of 30 frames per second. Data was analyzed by NTA 3.0 software (Malvern Instruments, Malvern, UK) on a frame-by-frame basis. The experiment was repeated four times.

### Co-culture of GSC-EXs with ECs

In order to determine whether GSC-EXs can be up-taken by ECs, the three types of GSC-EXs were labeled with PKH 26 and co-cultured with ECs [16]. In brief, GSC-EXs were labeled with PKH 26 (2 × 10^−6^ M, Sigma-Aldrich) for 5 mins at 37°C, followed by wash with 1 x PBS and ultracentrifuged at 170,000g for 90 mins. After that, GSC-EXs (50 μg) were resuspended and added to the culture medium (1 ml) of ECs for co-culture. After 24 hrs, ECs were washed with PBS for twice, and the nuclei of ECs were stained with DAPI (1 μg/ml, Wako Pure Chemical Industries Ltd) for 2 mins. Incorporation of GSC-EXs into ECs was observed by fluorescence microscopy (EVOS; Thermo Fisher Scientific). The level of cellular fluorescence intensity was analyzed by Image J (NIH) according to the instruction and a previous report [43]. Briefly, the cell of interest and a region next to the cell of interest that has no fluoresce (considered as background) were selected. The area, integrated density and mean gray value were measured. The cellular fluorescence was calculated as: cellular fluorescence = integrated density - (area of the selected cell x fluorescence of background readings). The fluorescence intensities of one hundred cells in five random fields in each group were averaged. The experiment was repeated four times.

To further elucidate whether GSC-EXs can alter the function of ECs, ECs were divided into five groups and co-cultured with: vehicle (co-culture medium only), GSC-EXs^con^, GSC-EXs^sc^, GSC-EXs^miR-21 ko^, GSC-EXs^miR-21^. The work concentration of GSC-EXs (50 μg/ml) was determined based on our previous study [16]. Some ECs were pre-transfected with VEGF siRNA (siRNA^VEGF^; 50 nM; Qiagen) using oligofectmanine transfection reagent (Invitrogen, Carlsbad, CA) for 48 hrs to knock out the endogenous VEGF, and then co-cultured with GSC-EXs^miR-21^. Scrambled siRNA (siRNA^sc^; 50 nM; Qiagen) was used as an experimental control. After 24 hrs, the culture medium of ECs were collected for VEGF analysis. The miR-21 level in ECs was analyzed by quantitative RT-PCR analysis. ECs were used for tube formation and migration assays. The experiment was repeated four times.

### Quantitative RT-PCR analysis

After transfection, cells were washed with 1xPBS. The total mRNA from GSCs was extracted using and lysed in Trizol (Thermo Fisher Scientific, Waltham, MA). The miRs from GSCs, and from ECs co-cultured with different types of GSC-EXs were extracted using mirVana miRNA Isolation kit (Qiagen). For detecting miR-21 level, reverse transcription (RT) reactions were performed by using mirVana qRT-PCR miRNA Detection Kit and hsa-miR-21 qRT-PCR primer set from Ambion. The forward primer of miR-21 was: 5′-TTTTGTTTTGCTTGGGAGGA-3′, the reverse primer of miR-21 was: 5′-AGCAGACAGTCAGGCAGGAT-3′. For measuring the mRNA level of VEGF in GSCs, RT was performed by using the first strand cDNA synthesis kit (Qiagen) according to manufacturer's instruction. The forward primer of VEGF was, 5′-CGAGGGCCTGGAGTGTG-3′, the reverse primer of VEGF was, 5′-CCGCATAATCTGCATGGTGAT-3′. The expression of U6 was used as endogenous control for each sample. The forward primer of U6 was: 5′-CTCGCTTCGGCAGCACA-3′, the reverse primer of U6 was: 5′-AACGCTTCACGAATTTGCGT-3′. Relative expression level of each gene was normalized to U6 and calculated using the 2^−ΔΔCT^ method. The experiment was repeated four times.

### Enzyme-linked immunosorbent assay

The level of VEGF in the culture medium of ECs co-cultured with the three types of GSC-EXs were measured by enzyme-linked immunosorbent assay (ELISA) according to the manufacturer's instructions (R&D Systems). The concentration of VEGF was calculated as pg/ml of culture medium. The experiment was repeated four times.

### Migration and tube formation assay for ECs

The migration assay was carried out by using the Boyden chamber (Chemicon, Rosemont, IL) as we previously reported with slight modification [44]. In brief, ECs (2 × 10^4^ cells) were seeded into the upper compartment of the boyden chamber. After 24 hrs, the ECs which migrated across the membrane were counted under an inverted light microscope. Ten random microscopic fields were assessed in each well. The average number of cells per field was determined.

The tube formation assay was conducted by using *in vitro* angiogenesis assay kit (Chemicon). First, the ECMatrix solution were thawed and mixed with the ECMatrix diluent. Then, the ECMatrix mixture were placed in a 96-well tissue culture plate at 37°C for 1 hr to allow the matrix solution to solidify. ECs (1 × 10^4^ cells/well) were seeded onto the solidified matrix and incubated at regular cell culture conditions (5% CO2, 37°C). After 24 hr post-seeding, 2 μg/ml calcein (Fisher scientific) was directly added to the culture well and incubated for 20 mins prior to imaging under an inverted fluorescence microscope. Tubes were defined as a tube structure exhibiting a length 4 times of its width [45]. Five random microscopic fields were assessed in each well. The average number of tubes per field was determined.

### Western blot analysis

After 24 hr co-culture, proteins of ECs in different groups were extracted with cell lysis buffer (Thermo Fisher scientific) supplemented with complete mini protease inhibitor tablet (Roche). Then, the protein lysates were electrophoresed through SDS-PAGE gel and transferred onto PVDF membranes. The membranes were blocked with 5% non-fat milk for 1 hr at room temperature, and incubated with primary antibody against VEGFR2 (1:1000; Santa Cruz), p-Flk1 (1:1000; Santa Cruz) or β-actin (1:4000; Sigma) at 4°C overnight. On the next day, membranes were washed and incubated with horseradish-peroxidase-conjugated anti-rabbit or anti-mouse IgG (1:40000; Jackson Immuno Research Lab) for 1 hr at room temperature. Blots were developed with enhanced chemiluminescence developing solutions and images were quantified under ImageJ software. The experiment was repeated four times.

### Statistical analysis

Data is expressed as mean ± SEM. Two group comparison was analyzed by Student's *t* test. Multiple comparisons were analyzed by one- or two-way ANOVA followed by LSD post-hoc test. SPSS 17.0 statistical software was used for analyzing the data. For all measurements, a *p* < 0.05 was considered statistic significant.
